# Supplementary material for: Empirical methods for controlling false positives and estimating confidence in ChIP-Seq peaks
Source: BMC Bioinformatics. 2008 Dec 5;9:523. doi: 10.1186/1471-2105-9-523 (PMC2628906; doi:10.1186/1471-2105-9-523)
Supplement: Additional File 1 — A variety of html documents from the USeq web site detailing the available applications, their best usage, output file type descriptions, command line menus, etc. [file 1471-2105-9-523-S1.zip › USeqUserGuides/JavaDoc.zip!/JavaDoc/util/bio/seq/IndexedSequence.html]

IndexedSequence


|  |  |  |
| --- | --- | --- |
| **Overview** \| **Package** \| **Class** \| **Use** \| **Tree** \| **Deprecated** \| **Index** \| **Help** | |  |
| **PREV CLASS**   **NEXT CLASS** | **FRAMES**    **NO FRAMES**     **All Classes** |  |
| SUMMARY: NESTED \| FIELD \| CONSTR \| METHOD | DETAIL: FIELD \| CONSTR \| METHOD |  |


---


## util.bio.seq Class IndexedSequence

```
java.lang.Object
  util.bio.seq.IndexedSequence
```

**All Implemented Interfaces:**: java.lang.Comparable

---

``` public class IndexedSequence extends java.lang.Object implements java.lang.Comparable ```

---

| **Field Summary** | |
| --- | --- |
| `static java.util.regex.Pattern` | `nameParser` |


**Constructor Summary**

`IndexedSequence(java.io.File binarySeqFile)`


| **Method Summary** | |
| --- | --- |
| `int` | `compareTo(java.lang.Object obj)`             Assumes starts are unique. |
| `boolean` | `contained(int basePosition)` |
| `java.lang.String` | `getChromosome()` |
| `java.lang.String` | `getSequence()` |
| `int` | `getStart()` |
| `int` | `getStop()` |
| `void` | `setSequence(java.lang.String sequence)` |

**Methods inherited from class java.lang.Object**

`equals, getClass, hashCode, notify, notifyAll, toString, wait, wait, wait`

**Field Detail**

### nameParser

```
public static final java.util.regex.Pattern nameParser
```


**Constructor Detail**

### IndexedSequence

```
public IndexedSequence(java.io.File binarySeqFile)
```


**Method Detail**

### compareTo

```
public int compareTo(java.lang.Object obj)
```

:   Assumes starts are unique.

    :   **Specified by:**: `compareTo` in interface `java.lang.Comparable`

---


### contained

```
public boolean contained(int basePosition)
```

---


### getChromosome

```
public java.lang.String getChromosome()
```

---


### getSequence

```
public java.lang.String getSequence()
```

---


### getStart

```
public int getStart()
```

---


### getStop

```
public int getStop()
```

---


### setSequence

```
public void setSequence(java.lang.String sequence)
```


---


|  |  |  |
| --- | --- | --- |
| **Overview** \| **Package** \| **Class** \| **Use** \| **Tree** \| **Deprecated** \| **Index** \| **Help** | |  |
| **PREV CLASS**   **NEXT CLASS** | **FRAMES**    **NO FRAMES**     **All Classes** |  |
| SUMMARY: NESTED \| FIELD \| CONSTR \| METHOD | DETAIL: FIELD \| CONSTR \| METHOD |  |


---
